# Magnetic Nanomaterials and Their Applications

**DOI:** 10.3390/nano4020505

**Published:** 2014-06-23

**Authors:** Yurii K. Gun’ko

**Affiliations:** 1 School of Chemistry and CRANN, Trinity College Dublin, Dublin 2, Ireland; E-Mail: igounko@tcd.ie; Tel.: +353-1-896-3543; 2 St. Petersburg National Research University of Information Technologies, Mechanics and Optics, St. Petersburg 197101, Russia

This Special Issue of *Nanomaterials* is dedicated to the development of new magnetic nanomaterials and their applications in biomedicine, catalysis, spintronics and other areas. The publications in this Issue demonstrate that the interest in magnetic nanomaterials is continuously growing and their realm is expanding rapidly. Some highlights of the publications in this issue are discussed below.

In their review article, Herranz *et al.* [1] present current achievements in the development of new magnetic nanomaterials for imaging, particularly for the *in vivo* detection of atherosclerotic plaques. The authors discuss different biofunctionalization techniques that have been recently developed. Special attention was paid to chemoselective techniques as these methods facilitate the application of magnetic nanomaterials in the clinic.

In their experimental work, Ota *et al.* [2] investigated anti-cancer cell effects using a combination of magnetic hyperthermia, an anti-Fas antibody and cryptotanshinone. The researchers have conjugated polyethylenimine coated magnetite nanoparticles to CH11 (an anti-Fas monoclonal antibody) and performed hyperthermia experiments on these nanoconjugates in cancer cells using an alternating magnetic field frequency of 210 kHz. It was found that HeLa cell growth decreased with increased doses of the antibody and complexes. Cell viability was also varied with the intensity of the applied alternating magnetic field. The introduction of cryptotanshinone (an anti-apoptotic factor blocker), also provided an additional anti-cancer cell effect [2].

Chen *et al.* [3,4] have published two papers on the properties of CoFeB/MgO/CoFeB and CoFeB/AlOx/Co Magnetic Tunnel Junctions (MTJs). In one paper, the MgO barrier layer thickness in CoFeB/MgO/CoFeB MTJs was varied to measure low-frequency alternate-current magnetic susceptibility and magnetic properties. It was found that the highest alternate-current magnetic susceptibility (χ_ac_) was obtained at a thickness of 11 Å, corresponding to an optimal resonance frequency of 10 Hz which should be useful for application in transformers, sensors, and magnetic read heads. It was suggested that the spin sensitivity is related to both highest magnetic susceptibility and maximum phase angle. Moreover in this work it was demonstrated that the indirect spin exchange coupling of top CoFeB and bottom CoFeB layers in CoFeB/MgO/CoFeB oscillates [3]. Similarly, in the other paper, the CoFeB/AlO*_x_*/Co multilayer film was used to investigate the strength of indirect spin exchange-coupling between CoFeB and Co strength and the χ_ac_ in a low-frequency alternating magnetic field, for various thicknesses of the barrier layer AlO*_x_* [4]. It was found that the maximum χ_ac_ value is obtained at the optimal frequency of 500 Hz. As the frequency increased further to 1000 Hz, the susceptibility rapidly declined to almost zero. It was established that the magnetic characteristics are related to the crystallinity of the Co and the thickness of the AlO*_x_* barrier layer. The multilayered magnetic tunnel junctions, with a high susceptibility in a low-frequency alternate-current magnetic field, are expected to find applications in low-frequency storage drives and magnetic recording media.

Rao *et al.* [5] prepared MgO films on Si substrates by direct current sputtering and investigated the effects of thermal annealing on the structural and magnetic properties of the films. It was found that there is a close correlation between room temperature ferromagnetism, crystallinity, and the magnesium vacancy concentrations in MgO thin films. The initial as-grown MgO films were amorphous due to the large lattice mismatch between MgO and Si substrate and demonstrated up to 9.62 emu/cm room temperature saturation magnetization values. However, after annealing, the films became nanocrystalline and the saturation magnetization values significantly decreased. The origin of the room temperature ferromagnetism has been explained by the presence of the magnesium cation vacancies. Thus, this research has shown an interesting approach to produce room temperature ferromagnetic MgO films and also helped to interpret the origin of ferromagnetism in MgO layes.

Finally, two papers on the use of magnetic nanomaterials in catalysis have been published by our group. Greene *et al.* [6] reported the synthesis, characterization and photocatalytic studies of cobalt ferrite-silica-titania core-shell nanostructures. In this work, the CoFe_2_O_4_@SiO_2_@TiO_2_ core-shell magnetic nanostructures have been prepared by the coating of cobalt ferrite nanoparticles with a double SiO_2_/TiO_2_ layer using metallorganic precursors. Then the core-shell nanoparticles were sintered at 600 °C to produce the photocatalytically active anatase phase at the surface of nanoparticles. It was found that these nanocomposites demonstrated a very efficient photocatalytic oxidation of methylene blue under UV light. The retention of magnetism in these core-shell nanostructures was of particular importance allowing the full magnetic recovery and re-use of the catalyst.

Govan *et al.* [7] reviewed the main recent advances in the development of magnetically recoverable nanocatalytic systems by the immobilisation of homogeneous catalysts onto magnetic nanoparticles. Particular attention was paid to magnetic core-shell nanostructures as substrates for catalyst immobilisation. The authors have also discussed binding of magnetic nanoparticles to inorganic catalytic mesoporous structures, catalytic metal organic frameworks (MOF), immobilization of catalytically active small organic molecules and polymers on magnetic carriers. In addition, some recent results on enzymatic catalysis using enzymes bound to magnetic nano-carriers have also been considered. The review has pointed out that very important progress was achieved in the development of various functional coatings on magnetic nanostructures including polymeric, dendritic, mesoporous zeolite-like and MOF coatings. The use of these functional coatings opens up new horizons in the development of new multifunctional catalysts with high reactivity and selectivity.

Overall, the papers published in this Special Issue represent important developments in the area of magnetic nanomaterials. We recommend you read through this Special Issue and look forward to seeing further progress in research on magnetic nanomaterials and their relevant applications.
